# MEG/EEG Source Reconstruction, Statistical Evaluation, and Visualization with NUTMEG

**DOI:** 10.1155/2011/758973

**Published:** 2011-03-15

**Authors:** Sarang S. Dalal, Johanna M. Zumer, Adrian G. Guggisberg, Michael Trumpis, Daniel D. E. Wong, Kensuke Sekihara, Srikantan S. Nagarajan

**Affiliations:** ^1^Department of Psychology, Zukunftskolleg, University of Konstanz, 78457 Konstanz, Germany; ^2^MEG Department, CERMEP, 69500 Lyon, France; ^3^INSERM U1028, CNRS UMR5292, Lyon Neuroscience Research Center, Brain Dynamics and Cognition Team, 69500 Lyon, France; ^4^Donders Institute for Brain, Cognition and Behaviour, Centre for Cognitive Neuroimaging, Radboud University Nijmegen, 6500 HB Nijmegen, The Netherlands; ^5^Sir Peter Mansfield Magnetic Resonance Centre, University of Nottingham, Nottingham NG7 2RD, UK; ^6^Division of Neurorehabilitation, Department of Clinical Neurosciences, University Hospital of Geneva, 1211 Geneva, Switzerland; ^7^Biomagnetic Imaging Laboratory, Department of Radiology and Biomedical Imaging, University of California, San Francisco, CA 94143, USA; ^8^Institute of Biomaterials and Biomedical Engineering, University of Toronto, Canada M5S 3G9; ^9^Department of Systems Design and Engineering, Tokyo Metropolitan University, Tokyo 191-0065, Japan

## Abstract

NUTMEG is a source analysis toolbox geared towards cognitive neuroscience researchers using MEG and EEG, including intracranial recordings. Evoked and unaveraged data can be imported to the toolbox for source analysis in either the time or time-frequency domains. NUTMEG offers several variants of adaptive beamformers, probabilistic reconstruction algorithms, as well as minimum-norm techniques to generate functional maps of spatiotemporal neural source activity. Lead fields can be calculated from single and overlapping sphere head models or imported from other software. Group averages and statistics can be calculated as well. In addition to data analysis tools, NUTMEG provides a unique and intuitive graphical interface for visualization of results. Source analyses can be superimposed onto a structural MRI or headshape to provide a convenient visual correspondence to anatomy. These results can also be navigated interactively, with the spatial maps and source time series or spectrogram linked accordingly. Animations can be generated to view the evolution of neural activity over time. NUTMEG can also display brain renderings and perform spatial normalization of functional maps using SPM's engine. As a MATLAB package, the end user may easily link with other toolboxes or add customized functions.

## 1. Introduction

As exemplified by this special issue on open-source analysis toolboxes, many software solutions exist to suit a variety of experimental goals and level of end-user programming experience, including the option to mix and match toolboxes for different stages of processing. However, a decade ago, few options existed for analyzing magnetoencephalography (MEG) data with noncommercial open-source software, especially for more sophisticated inverse algorithms or with a graphical interface to navigate results.

Electroencephalography (EEG) analysis and corresponding software packages are dominated by sensor level processing, such as topography, evoked responses, and ICA. Source localization is more feasible with MEG data; however, many commercial packages offer only one of several basic inverse methods (dipole fitting, beamforming, and minimum-norm). Within open-source options available at present, BrainStorm (http://neuroimage.usc.edu/brainstorm) and MNESuite (http://www.nmr.mgh.harvard.edu/martinos/userInfo/data/sofMNE.php) offer similar source localization options; FieldTrip (http://fieldtrip.fcdonders.nl/) additionally offers beamforming, and SPM8 (http://www.fil.ion.ucl.ac.uk/spm/) offers an advanced Bayesian source estimation method. However, to date, other packages do not provide a whole suite of reconstruction algorithms ranging from the simple to the complex powerful ones that have been recently published.

In 2003, the seeds of NUTMEG (Neurodynamic Utility Toolbox for Magnetoencephalo- and Electroencephalo-Graphy) were planted at the University of California, San Francisco (UCSF), with the motivation to meet several research goals, including implementation of experimental source localization algorithms and general independence from commercially provided software, as well as user extensibility for custom analyses [1]. Specific strengths of NUTMEG include: (1) choice of several inverse algorithms, including variants of popular beamforming, minimum-norm, and Bayesian inference techniques, (2) intuitive viewing and navigation of results, (3) both GUI and command-line batch use, and (4) several methods of source space functional connectivity analysis.

NUTMEG can be downloaded from http://nutmeg.berkeley.edu/. Documentation and a user's wiki are also located at this website, and users can subscribe to a mailing list which is intended as a general forum for questions related to the software itself or analysis procedures.

NUTMEG is primarily written in MATLAB (MathWorks, Natick, MA, USA). The MATLAB Signal Processing Toolbox is required for digital filter operations, and the Image Processing Toolbox is needed for (optional) graphical volume-of-interest (VOI) selection. A link with SPM8 allows activations to be overlaid onto standard orthogonal magnetic resonance imaging (MRI) slices or a rendered 3D brain volume; at present, SPM8's data analysis engine is not used. Via SPM8, activations may also be spatially normalized and displayed on an MNI template brain [2, 3]. Visualization tools in Python (http://www.python.org/) are under development and will be made available in future versions.

NUTMEG is also interoperable with other software for, for example, scrolling through and artifact rejection of sensor data (FieldTrip and Brainstorm), BEM forward models (OpenMEEG, http://openmeeg.gforge.inria.fr/), FieldTrip, SPM8, Helsinki BEM Toolbox), preprocessing (CTF MEG software, MEG International Services, Coquitlam, Canada), and ELAN (see [4]).

## 2. Philosophy

### 2.1. Need for Open Source

Releasing analysis software as open source provides a fair and effective means to distribute methodological developments made possible by public research funds, as well as to promote the spirit of academic scientific cooperation. Additionally, the open-source model allows the same analysis methods to be easily used with nearly any type of input data, regardless of equipment manufacturer. The source code, being open to the end user and the academic community at large, also becomes a transparent tool, removing any mystery as to how data is being manipulated and allowing custom modifications; any errors can be found and corrected more efficiently as well. Furthermore, both the theory and practical implementation of analysis methods for MEG/EEG data have been rapidly developing in the past two decades. Whether one is a methods researcher comparing algorithms or a cognitive scientist eager to use the latest methods, neither should have to wait the several years that it can sometimes take for a method to be released as part of a proprietary software package.

### 2.2. Types of Data/Experiments/Paradigms

Other functional neuroimaging modalities such as fMRI benefit from a relatively established stream of standard processing steps that facilitate learning by beginners and batch processing by more experienced users. However, with MEG and EEG, it sometimes seems that there can be as many ways of analyzing the data as there are datasets, as appropriate analyses can vary considerably according to the paradigm and the types of responses. A useful software package needs to be flexible enough to introduce the various analysis streams as they are developed and straightforward to use for routine analysis.

Experiment types that have been successfully processed with NUTMEG include (1) evoked paradigms, for example, auditory stimulation in healthy subjects [5], verbal stimulation compared between healthy subjects and schizophrenia patients [6], perturbation of self-speech perception [7], and somatosensory stimulation in humans and monkeys [8], (2) time-frequency analysis, for example, finger movements [9], visual stimulation [10], decision making [11], discrimination of tone rate modulation [12], and visually guided behavior [13], and (3) resting state and task-induced connectivity [14, 15]. Data types supported in NUTMEG include MEG, EEG [16], and intracranial EEG [17].

### 2.3. Integration with Other Toolboxes

It is logical for certain basic software components, such as data import/export, to be shared between different toolboxes. Nevertheless, a particular software package may excel for certain processing or analysis procedures; it would benefit other software packages to be able to call the code for such components transparently from within their own package. Throughout the description of processing steps for NUTMEG, we will describe which procedures are specifically implemented in NUTMEG and which ones take advantage of links to other software.

## 3. NUTMEG Processing Steps

**Figure 1:**
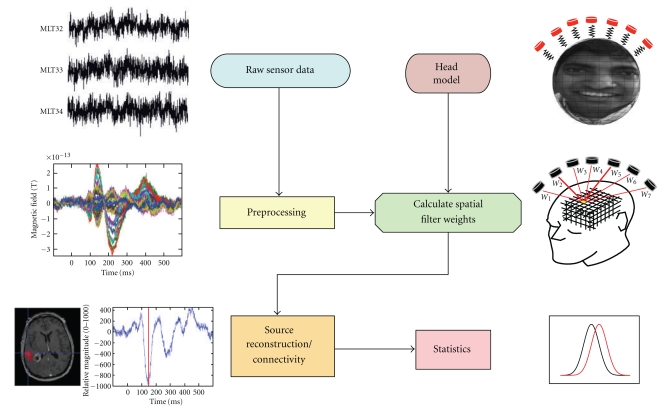
NUTMEG workflow. After preprocessing, sensor data is combined with head coregistration information to create a forward lead field model, which is then used to compute a source estimate either for time-series or time-frequency domains. Source space data can be further processed with single- or multisubject statistics, or for functional connectivity.

The first step of the NUTMEG workflow (illustrated in Figure 1) involves loading in the MEG/EEG data and, if available, MRI and coregistration information. A forward lead field is computed within NUTMEG, or imported from external software. This information is all stored in a MATLAB structure that advanced users may access from the command line or with user-created scripts, facilitating links with other software. Translators between NUTMEGs structure and the FieldTrip and ELAN formats are included in the standard NUTMEG distribution. Likewise, results are stored in a separate structure, so that derived outputs such as “virtual electrode” time series can be further analyzed in MATLAB with the user's preferred tools. Results from external programs can also be reformatted to allow viewing with NUTMEGs results navigator.

**Figure 2:**
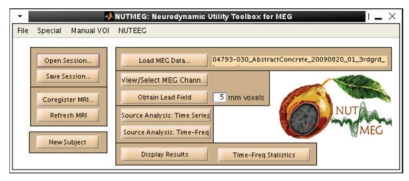
NUTMEG session main interface for guiding the user through processing steps and calling other GUIs for loading data and coregistration and computing forward lead field and source localizations.

The main GUI (Figure 2) guides a new user through the processing steps, greying out the boxes that cannot yet be completed given the information currently provided. An advanced user may bypass the GUI and operate all steps from the command-line.

### 3.1. Loading Different Data Types

NUTMEG can import MEG, EEG, and intracranial EEG data from various manufacturer's systems. At present, this includes CTF, 4D/BTi, KIT/Yokogawa, and Elekta Neuromag MEG systems, as well as EEG data from BrainProducts and Micromed. Several other formats are also supported via a link with the *fileio* module of FieldTrip. Data may comprise unaveraged multiple trials, an average across trials, or continuous data.

### 3.2. Sensor Preprocessing

**Figure 3:**
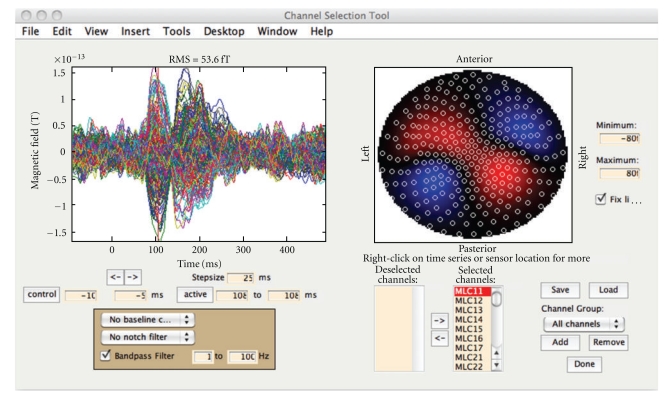
Sensor Preprocessing GUI. The left subplot shows the averaged time series for each sensor overlaid. The right subplot shows the RMS over a selected time window. The effects of filtering and time window selection on both the sensor time series and RMS spatial plots can be viewed here. Channels may be (de-)selected for further processing, either as individual noisy channels or groups of channels by spatial region.

After loading the data into NUTMEG, the user may click on “View/Select MEG Channels” from the main GUI (Figure 2), which opens a new window (Figure 3). After selecting a time window of interest, the root mean square of the sensors is displayed on a 2D sensor map. Sensors can be (de-)selected for further processing. The effects of baseline removal and filtering on the sensor map can also be examined.

Preprocessing components from other software packages may optionally be used as well and imported into NUTMEG. SPM8 has especially useful tools for automated artifact rejection. For more advanced sensor or trial selection, the graphical interface from FieldTrip could be used.

### 3.3. Forward Methods

**Figure 4:**
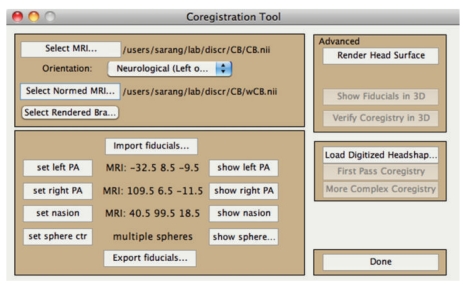
Coregistration Toolbox GUI allows inclusion of subject's MRI, fiducials to be loaded or set manually, spatially normalized MRI or rendered surface (created via SPM8), surface mesh, and digitized headshape points.

NUTMEG includes a built-in single sphere [18] and multisphere model for MEG [19]. The individual subject's structural MRI or digitized headshape can be loaded via the Coregistration Tool GUI (Figure 4). Here, additional information such as a spatially normalized MRI or rendered brain surface (created via SPM8) can be loaded, and fiducial positions can be imported or manually set. Furthermore, a head surface mesh can be generated within NUTMEG, which can aid with fiducial coregistration if digitized headshape measurements have been made, for example, with a Polhemus FASTRAK device (Colchester, VT, USA). If no individual subject MRI or headshape is available, the MNI template brain may be used in their place. Cortical segmentation is not used to constrain either the source locations or orientations computed within NUTMEG, as slight errors in coregistration may lead to larger errors in source estimation. Lead fields for scalp and intracranial EEG can be computed within NUTMEG, currently implemented as a simple semi-infinite homogeneous volume conductor.

Additionally, boundary element model (BEM) and finite element model (FEM) head models can be generated externally and imported for use with NUTMEG for either MEG or EEG. Currently supported external models include OpenMEEG (BEM), MNE (BEM), FieldTrip (BEM), and SMAC [20] (spherical model with anatomical constraints). A link to generate and import FEM from SimBio/NeuroFEM (https://www.mrt.uni-jena.de/simbio) is planned. Imported lead fields may be specified either with free orientations in vector form or orientation-constrained in scalar form.

After the data and coregistration information are loaded and lead field obtained, the NUTMEG main GUI (Figure 2) will make available the buttons for source estimation.

The coregistration from MEG sensors to MNI coordinates can also be used independently of NUTMEGs source localization tools in order to obtain MNI coordinates of dipole fits computed elsewhere, as shown in Zhu et al. [21].

### 3.4. Inverse Methods

**Figure 5:**
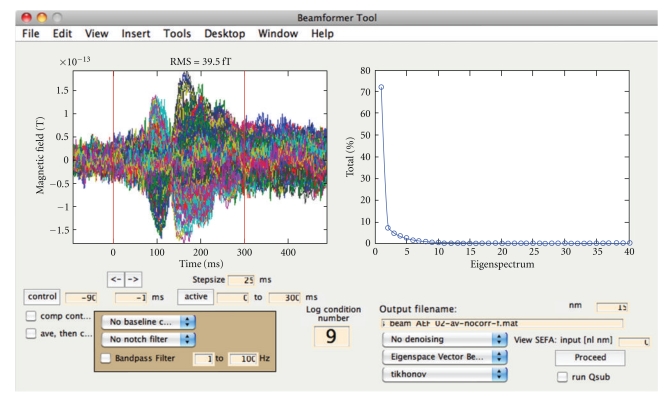
Time-series source estimation GUI. The left subplot shows the averaged sensor time series for each sensor. The right subplot changes depending on user selection. The eigenvalues can be plotted (as shown) for assisting the eigenspace beamformer; alternatively, cleaned sensor data and factors can be plotted if SEFA denoising is selected. The condition number of the sensor data covariance is displayed to help prevent meaningless results computed with overly high condition numbers. Other options here include filtering, time window selection, averaging before/after data covariance computation, method of source inversion, other denoising methods, sensor covariance regularization, and the option to submit the computation to a grid cluster for site-specific setups.

NUTMEG can be used to localize evoked (averaged) data or induced (nonphase-locked) data. Certain methods are better tuned to each type of analysis. When the user clicks the button from the main GUI (Figure 2) called “Source Analysis: Time-Series,” a new window appears (Figure 5). Included in this window are drop-down menus for choice of inverse method and regularization. The user has a choice about whether to use the covariance from averaged or single-trial data, for those inverse algorithms that use data covariance. The main GUI has tick-boxes for this averaging choice, for whether to create a contrast with a control time window, and for whether to send the computations to the “qsub” distributed job manager.

#### 3.4.1. Beamformers

The most commonly used and developed class of inverse method within NUTMEG is the beamformer. This is an adaptive method which minimizes the variance at a given source location while suppressing noise from other locations [22]. It takes as inputs both the sensor data covariance and the forward lead field, represented by the basic formula

(1) $$\begin{array}{c} W_{r}^{T}=\frac{{L_{r}}^{T}R_{yy}^{-1}}{{L_{r}}^{T}R_{yy}^{-1}L_{r}}, \end{array}$$

where **W**_*r*_ comprises the computed sensor weights to derive the activity at brain location *r*, **L**_*r*_ contains the gain at each sensor (forward model) for a source at location *r*, and **R**_*yy*_ is the sample covariance for the chosen data segment.

Many flavors of beamforming are created by the many ways to compute the data covariance estimate and lead field. These choices can be dictated by the experimental paradigm or by tradeoffs of computational intensiveness versus accuracy (in the case of a lead field). The data covariance estimate needs to be optimally tuned to the effect of interest (e.g., time window length and filter parameters) while maintaining invertibility. The sample data covariance may be computed either from an averaged evoked response or by averages of the sample covariance of each trial, as selected by the user with the tick-box on the Beamforming Tool GUI (Figure 5); also see section Regularized Beamformer for Evoked Data.

#### 3.4.2. Eigenspace Beamformer for Evoked Data

Sekihara et al. [23] proposed the eigenspace beamformer to improve stability of reconstructions using averaged evoked responses. Based on the singular value decomposition (SVD) of the covariance matrix of the averaged data, the user defines the *signal space* from the largest few eigenvalues, rejecting eigenvalues from the remaining *noise space*. A signal space data covariance is then computed and inverted, replacing the data covariance of the weight formula (1) in the numerator while maintaining the original evoked response covariance in the denominator. This method has the advantage of improving weight computation for averaged data, focusing the result on the eigenvectors of interest, and allows for effective removal of large-amplitude phase-locked artifacts. To aid selection of desired signal space eigenvalues, the right-hand plot within the “Source Analysis: Time-Series” GUI (Figure 5) displays the relative magnitudes of the eigenvalues and the time course of the top selected eigenvectors.

#### 3.4.3. Time-Frequency Beamformer

**Figure 6:**
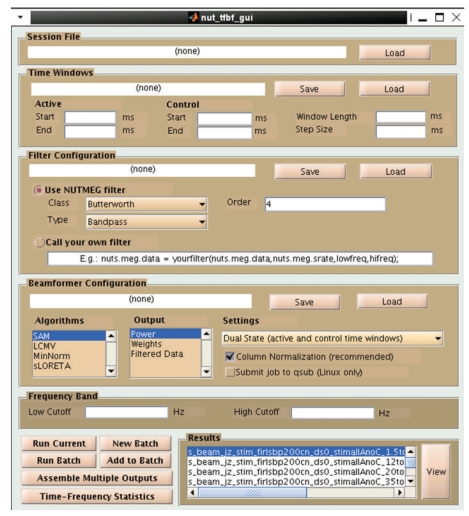
Time-Frequency Beamformer GUI. The pre-specified NUTMEG session including dataset and forward lead field is loaded. Multiple active windows and a single control time window, as well as filtering parameters are selected in the GUI or loaded from prespecified parameter files. Details of the source inversion methods are specified, and the whole setup can then be saved and set to run as a batch process, potentially sending each time-frequency window to a separate computer node. Finally, all the windows are recombined into one file to view the 5-D output.

**Figure 7:**
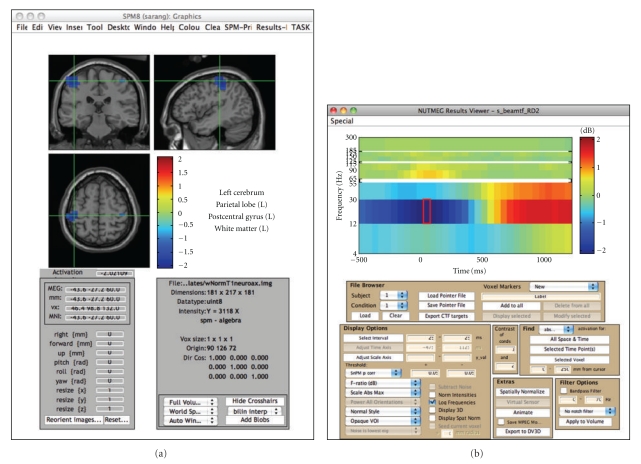
NUTMEGs results viewer, in time-frequency mode. The modified SPM8 viewer shows the functional map corresponding to the selected time-frequency window, marked by a red box in the spectrogram. Conversely, the time-frequency spectrogram corresponds to the voxel indicated by the cross-hairs on the MRI navigator. The MRI display and time-frequency spectrogram are interactively linked to each other; changing a selection in one automatically updates the other. Movies can also be created from animations of the functional images across time.

Dalal et al. [9] developed a method for optimized time-frequency beamforming (TFBF). NUTMEG implements this algorithm to be computed easily over a grid of many time-frequency windows, assembling the results for intuitive interactive navigation (Figures 6 and 7(b)). TFBF is based on the LCMV beamformer [22] and contrasts each active time-frequency window with a common control window. The user is encouraged to select time windows as short as possible to focus on transient and frequency-specific power changes, within the confines of SNR and period of oscillation for the given frequency band. Data covariance for TFBF is estimated by averaging the sample covariance from each trial.

The TFBF GUI (Figure 6) is opened from the main GUI by clicking “Source Analysis: Time-Freq” and guides the user through selecting options. These parameters can be saved and called again or run as a batch process on a single computer or a high performance computing grid.

#### 3.4.4. Regularized Beamformer for Evoked Data

SAMerf and erSAM [24] use weights derived from the data covariance of unaveraged data and applied to evoked averaged data; however, these may not be optimally tuned for phase-locked activity, especially if the nonphase-locked activity is stronger. To overcome the difficulty of inverting the ill-conditioned matrix obtained from the covariance of averaged data, Brookes et al. [25] proposed to regularize with the minimum eigenvalue of the unaveraged data covariance. This option is included in the “regularization-type” drop-down menu on the Source Analysis: Time-Series GUI (Figure 5) and can be used with the Scalar LCMV Beamformer applied to averaged data. This option works especially well for stimulus-driven phase-locked effects, such as from a flashing checkerboard.

#### 3.4.5. Coherent Source Suppression

An occasional point of failure with beamformer techniques occurs when two sources are highly temporally correlated, as might occur, for example, in some subjects with bilateral auditory evoked responses. We have implemented a *coherent source suppression* technique in NUTMEG that can overcome such a correlated source failure [5]. A zone containing an expected interfering source must be defined, and this can be accomplished interactively with the MRI viewer. The algorithm has been independently shown to improve upon standard beamformer performance whether using the “partial sensor coverage” strategy [26] or using whole head coverage as usual [27]. The method has also been successfully applied to suppress cochlear implant artifacts in EEG data [16].

#### 3.4.6. Source Stability Index for Evoked Data

Another method to bypass the problem of beamformers with temporally correlated evoked sources is proposed by Prendergast et al. [28], termed the *Source Stability Index*. First, one obtains a source localization estimate from data covariance of unaveraged trials, then the corresponding weights are applied to an average of two separate halves the trials. The correlation at each voxel between the source estimates derived from the separate halves will be high at locations of a true evoked source; this step is repeated for different divisions of the trials, and an average correlation map is obtained to localize the sources. This method has been implemented to work within the NUTMEG work flow and has been successfully applied to auditory evoked data from tone stimulation [29].

### 3.5. Bayesian Inference Inversions

The following denoising and source localization methods are designed to be used with averaged data. If unaveraged trials are loaded, they will be averaged first prior to input into these methods.

#### 3.5.1. Denoising/Factor Analysis of Sensor Data

To remove background noise from evoked data with a prestimulus baseline, Nagarajan et al. [30] proposed *stimulus evoked factor analysis* (SEFA). The SEFA algorithm uses Bayesian inference to determine which temporal “factors” (like a component in ICA) are stimulus-evoked versus background activity. Using a probabilistic model, hyperparameters over each factor help determine which to keep or to suppress. SEFA can be selected from the drop-down “denoising” menu, and the immediate effects on the cleaned sensor data can be viewed within a subfigure of the Source Analysis: Time-Series GUI.

#### 3.5.2. SAKETINI Inverse Method

In order to estimate source activity using knowledge of event timing and independent from noise and interference (SAKETINI), a probabilistic model [31] was proposed which, for each source voxel, separates the contribution to the sensors from (1) evoked activity at that given voxel, (2) evoked activity at all other voxels, (3) background neural activity present in the prestimulus period, and (4) sensor noise. Using a similar probabilistic model to SEFA but with an additional term for (1), SAKETINI also uses hyperparameters to determine how many factors belong to each category. This method can be called from the drop-down menu on the Source Analysis: Time-Series GUI (Figure 5) or from batch scripts and can optionally be run on a parallel computing cluster to speed computation time.

#### 3.5.3. NSEFALoc Inverse Method

Using the factors from SEFA as a set of temporal basis functions (TBFs), the neural SEFA localization (NSEFALoc) method [32] determines, for each source voxel, the optimal linear combination of the TBFs with an additive noise term to model voxels at which no evoked activity occurs. NSEFALoc has been shown to be superior to the eigenspace beamformer and minimum-norm methods for evoked activity. Like SAKETINI, it can be called from the GUI or command line and may also optionally be run on a parallel computing cluster.

#### 3.5.4. Champagne Inverse Method

NUTMEG also implements Champagne [33], a tomographic Bayesian inference algorithm that combines SEFA modeling of background noise with sparse Bayesian inference of source activity in all voxels simultaneously using fast, robust update rules with guaranteed convergence under many realistic conditions. Champagne bears some similarities to SAKETINI. Whereas SAKETINI considers each voxel sequentially while statistically modeling contributions to sensors from other voxels, Champagne considers all voxels simultaneously. Champagne has been shown to successfully localize many simultaneous and temporally correlated sources.

### 3.6. Minimum-Norm Methods

For algorithm performance evaluation as well as comparison with results from the literature, two common minimum-norm methods are also included in NUTMEG. Both sLORETA [34] and dSPM [35] normalize the standard mininum-norm inverse

(2) $$\begin{array}{c} L^{T}{\left(LL^{T}\right)}^{-1} \end{array}$$

by an estimate of source noise obtained by projecting sensor noise

(3) $$\begin{array}{c} L^{T}{\left(LL^{T}\right)}^{-1}R_{nn}{\left(LL^{T}\right)}^{-1}L. \end{array}$$

By using a form of the regularized Gram matrix **L** **L** ^*T*^ in place of **R**_*yy*_ in (1), the (data-dependent) beamformer can be translated to a (data-independent) weighted minimum-norm method. As the Gram matrix is not full-rank, yet needs to be inverted, performance is highly dependent on choice of regularization. NUTMEG includes two options to regularize the Gram matrix prior to inversion: (1) add the sensor covariance matrix (based on either individual subject data or room noise) weighted by a constant or (2) Tikhonov regularization, that is, add a constant to the diagonal of the Gram matrix, based on the strength of the off-diagonal elements of the inverted matrix.

dSPM traditionally sets **R**_*nn*_ to room noise covariance. If this covariance is taken to be the identity matrix (times a constant), this leads to the regularized Gram matrix in the numerator in place of **R**_*yy*_ and the square of the regularized Gram matrix in the denominator. In contrast, sLORETA sets **R**_*nn*_ to the sensor noise covariance obtained from assuming (in a Bayesian fashion) identity source power and identity sensor noise (times a constant), which is equivalent to a type of regularized Gram matrix.

Note that, while minimum-norm spatial filters are non-adaptive relative to the sensor data, they enforce that all measured activity arises from the defined VOI. A cortically constrained VOI is often used with both methods.

Lastly, NUTMEG implements a recently developed method by Kumihashi and Sekihara [36] called the *Array Gain constraint Minimum-Norm, with Recursively Updated Gram matrix* (AGMN-RUG) method, which estimates the sensor covariance matrix by recursively updating the weighted Gram matrix using the source covariance from the previous estimate. Like the minimum-variance adaptive beamformers, the source estimates are spatially focal, while, like the minimum-norm methods, unhindered by temporally correlated sources or few available time points.

## 4. Visualization

NUTMEG supports both orthogonal view visualization as well as 3D rendering of cortical surface visualization. NUTMEG utilizes the SPM8 navigator to display functional maps on structural MRIs (Figure 7(a)). This is interactively linked with NUTMEGs time series or time-frequency display (Figure 7(b)). That is, when the user clicks to a different location in the brain, the time series/frequency display updates to show the temporal change at that location. Likewise, the user can click on a different time point or frequency band and the MRI display automatically updates with the 3D functional map corresponding to that new time/frequency point. Additional buttons exist for manipulating the view, for example, displaying different contrast types (simple difference of active and control, % change, etc.), zooming in time, rescaling the colormap, and calling SPM8 functions for projecting functional overlays onto a rendered surface.

In addition to these main tools, data can be exported to analyze format images which can then be further manipulated in CarTool (http://sites.google.com/site/cartoolcommunity/), mri3dX (http://www.cubric.cf.ac.uk/Documentation/mri3dX/), DataViewer3D (https://www.ynic.york.ac.uk/software/dv3d), and MRICro (http://www.cabiatl.com/mricro/), all of which can be used to generate publication-quality surface renderings with superimposed functional maps.

## 5. Statistics

### 5.1. Within-Subject Statistics

A nonparametric statistical threshold for time series source reconstructions can be calculated based on the distribution of baseline activity across trials within a single subject [37]. For time-frequency source reconstructions, Wilcoxon *Z* scores assess the contrast between baseline time-frequency windows versus “active” windows [17].

### 5.2. Group Statistics

Group statistics can also be performed to assess statistical significance across subjects. The mean and variance of power across subjects can be computed by first spatially normalizing each subject's source reconstruction and then resampling each subject's result into a common voxel space.

Statistical tests can then be applied to these transformed datasets. For situations in which normal distributions of power change can be expected, or after transformation to a normal distribution, one option is to apply the Student's *t*-test or ANOVA across multiple conditions.

Alternatively, statistical nonparametric mapping (SnPM) can be applied to data that may not necessarily follow a normal distribution [38]. One of the advantages of SnPM over parametric methods is that it can be applied to a population of as few as 5 subjects, though having more subjects will allow detection of weaker effects. Since variance estimates can be noisy for a relatively low number of subjects, variance maps are smoothed with a 3D Gaussian kernel. From this, a pseudo-*t* statistic can be obtained at each voxel, time window, and frequency band. Then, a distribution of pseudo-*t* statistics is created from 2^*N*^ permutations of the original *N* datasets (subjects). Each permutation consists of two steps: (1) inverting the polarity of the power change values for some subjects (with 2^*N*^ possible combinations of negations) and (2) finding the current maximum pseudo-*t* value among all voxels and time windows for each frequency band. Instead of estimating the significance of each nonpermuted pseudo-*t* value from an assumed normal distribution, it is then calculated from the position within the distribution of these maximum permuted pseudo-*t* values. The comparison against maximum values effectively corrects for the family-wise error of testing multiple voxels and time windows.

## 6. Connectivity

The brain is a complex network with abundant functional interactions among local and remote brain areas [39]. The synchronization of oscillations in different brain areas, that is, the so-called *functional connectivity*, is considered as an index of their functional interaction [40, 41]. Techniques based on functional connectivity open an accessible window for a noninvasive assessment of brain function in healthy subjects [42, 43] as well as in patients with brain lesions [14, 44].

NUTMEG computes the localization of functional connectivity among brain areas from MEG and EEG recordings by combining source localization algorithms with measures of functional connectivity. The oscillations of neural networks at each brain voxel are estimated by calculating the linear combination of the sensor data matrix with a spatial weighting matrix obtained with inverse solutions.

### 6.1. FCM Toolbox for Imaginary Coherence

**Figure 9:**
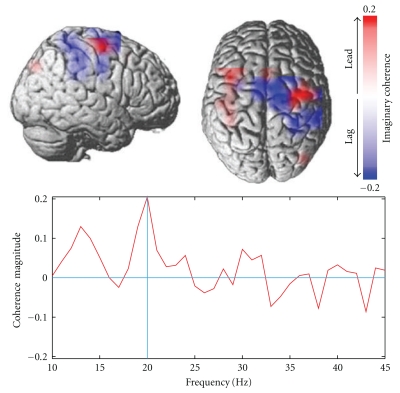
Corticomuscular coherence, showing relationship between motor and somatosensory cortices and left finger EMG. Note that regions that lead and lag the EMG activity can be clearly differentiated.

Imaginary coherence, applied to the source time series, is a measure of functional connectivity that is robust to sensor cross-talk and volume conduction [14, 45]. In order to reduce computation times for large datasets or for exploration of numerous connections among brain voxels, the calculations in NUTMEG can be performed in parallel on Linux clusters. The toolbox also offers visualization tools for inspection of the complex functional interactions data as well as a set of statistical tests.  Figure 9 shows an example of corticomuscular coherence in a single subject, which is localized to the bilateral motor cortex. Cortico-cortical interaction can also be analyzed.

### 6.2. Full Coherence

**Figure 10:**
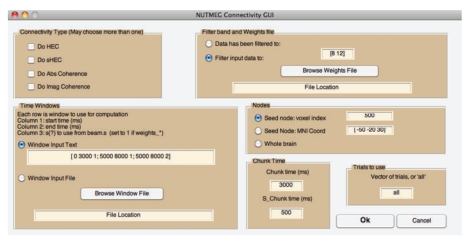
The connectivity GUI allows the user to specify the type of metric, the frequency band of interest, the whole time windows of interest which may be across condition types, the length of time chunk within each time window, and whether to compute over whole brain or seed based. The GUI assumes the inverse weights have already been computed, but the source-level time series or power need not have been saved out previously.

As a standalone command line option or called from a GUI (Figure 10), both the magnitude and imaginary cross-coherence can be computed for an input of voxels' power spectrum (after FFT and windowing of time series) for each trial. The output can be placed into the appropriate NUTMEG data structure to view results overlaid on the MRI.

### 6.3. Hilbert Envelope Correlation

An alternative metric for MEG/EEG functional connectivity involves computing the correlation of the Hilbert envelope (amplitude) of bandpass filtered time series from source locations [46]. This method may also be called from a GUI (Figure 10) or command-line.

## 7. Extension to Include Scalp and Intracranial EEG

### 7.1. Scalp EEG

**Figure 11:**
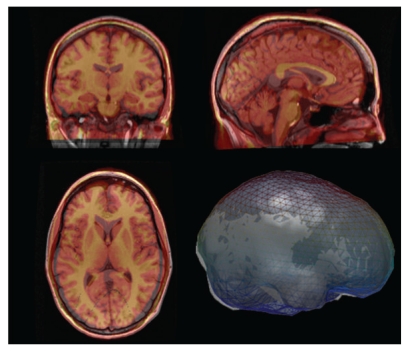
The overlay of the warped template MRI (red/yellow) with the actual MRI (grey) shows a reasonable fit of the scalp surface and neuro-anatomy. A mesh overlay of the warped template brain (blue) and the actual brain (grey) is also shown.

NUTMEG has been expanded to support beamforming with electroencephalography data via the NUTEEG module. This module allows the import of data recorded from EEG systems, along with electrode coordinates. NUTEEG automatically performs average referencing on EEG data and lead potentials as part of the preprocessing procedure. For situations where an MRI is not available, NUTEEG provides the option of warping a template MRI and corresponding boundary element model to digitized electrode positions, based on the algorithm described by Darvas et al. [47] (see Figure 11).

**Figure 12:**
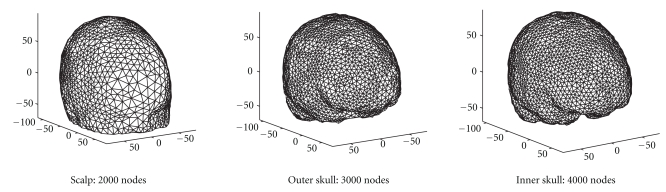
Three-layer boundary element mesh created with Delaunay triangulation. Projected electrode positions are shown as blue dots on the scalp surface.

Forward lead potentials can be calculated either using spherical head models, or via BEM with the previously mentioned toolboxes. If a boundary element model is used, digitized electrode positions can be projected to the scalp surface. Boundary element models can be created from segmented MRI images using a Delaunay triangulation method provided by the ISO2MESH toolbox (http://iso2mesh.sourceforge.net/) (Figure 12), or from BrainSuite Duff surface files via a triangulated sphere wrapping procedure.

**Figure 13:**
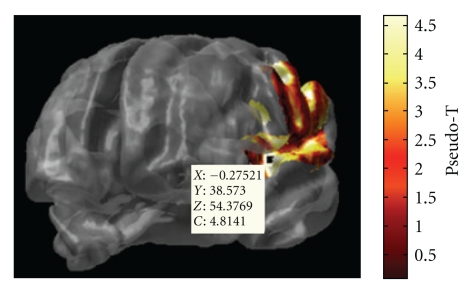
EEG beamformer reconstruction of an evoked response from auditory cortex projected to the cortical surface.

NUTEEG allows the user to import cortical surface Duff files from BrainSuite to create a file containing orthogonal dipole orientations for voxels near the cortical surface. These dipole orientations can then be used for implementing cortical constraints, where one assumes that sources are cortical and are oriented tangential to the cortical surface. The imported cortical surface files can also be used to show results in 3D (Figure 13).

After data import and lead field computation/import of EEG, the subsequent steps for source estimation and visualization are straightfoward, as for MEG.

### 7.2. Intracranial EEG

Invasive electrode implants are sometimes performed in human patients to aid in surgical planning for, for example, intractable epilepsy or brain tumors. Although intracranial EEG is often considered to be the “gold standard” of electrical brain activity, it may also be susceptible to undesired physiological noise sources [48, 49]. Furthermore, intracranial electrodes are not immune to far-field potentials from strong brain sources.

Referencing choice can also complicate interpretation of results. A simple focal source appears as a polarity inversion between electrodes in a monopolar scheme but a local peak in a bipolar montage. Furthermore, for complex voltage topographies, the actual source origin may be ambiguous and difficult to deduce from any montage. Finally, traditional voltage topographies are limited by the spatial sampling of the electrode placement.

Source localization techniques from scalp EEG/MEG may provide a solution to these problems. In particular, adaptive spatial filtering methods such as beamforming are particularly well suited [50]. Unlike previous attempts that use minimum-norm-based techniques [51–54], beamformers do not enforce that all source activity arise from the defined volume of interest. Thus, noise sources such as heart and muscle would be rejected by the spatial filter rather than projected into the brain, and, conversely, brain regions that contribute negligible signal would not distort the localization results. Finally, source localization allows gaps between electrodes to be “filled in” to gain an effectively higher spatial resolution, providing similar benefits to denser electrode coverage.

**Figure 14:**
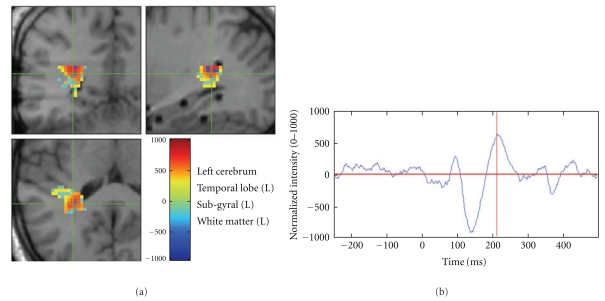
Intracranial EEG beamformer reconstruction of a visual evoked response. Depth electrode trajectories are evident on the sagittal MRI view.

Therefore, development of intracranial EEG localization and analysis techniques is considered a research priority for NUTMEG.  Figure 14 shows preliminary results from a beamformer applied to depth electrode responses evoked by photographic stimuli. Lead fields can be computed within NUTMEG, currently implemented as a simple semi-infinite homogeneous volume conductor; alternatively, a BEM-based lead field can be computed and imported from the OpenMEEG package.

## 8. NUTMEG in Python

Python is an open-source, general-purpose, object-oriented programming language that is gaining popularity as a tool for scientific computing. As an interpreted language with robust object model support, Python allows a wide variety of programming styles, from line-by-line scripting to abstracted, reusable library code. Its strengths include an emphasis on legibility and ease-of-use, system portability, and straightforward access to system libraries. Additionally, there is a very stable stack of basic computational tools actively developed by the scientific Python community. First among the many commonly used tools are: NumPy for multidimensional arrays, SciPy for a wealth of computational code, much of it being a Python layer over established, validated libraries such as LAPACK and FFTPACK, and Matplotlib, which provides interactive and scriptable 2D plotting tools that emulate MATLAB plotting. All these features provide a convenient computing environment for the development of modern scientific data processing systems, whose scope may expand over time, and whose core functionality typically demand a design covering a range from optimized algorithms to complex data models for physical phenomena.

NUTMEG-Py is a complementary project that entails a small scale reformulation of NUTMEG components into Python. To date, implementation of visualization and statistical postprocessing have been emphasized, with source reconstruction algorithms remaining in MATLAB.

### 8.1. From MATLAB Data to Python Objects

The workflow for a NUTMEG-based analysis that incorporates Python tools presents both a design challenge and a technical data translation problem. The latter is a solved problem, thanks to code from SciPy enabling I/O between NumPy arrays and MATLAB data contained in MAT files. The former allows the use of Python's object model.

NUTMEG-Py's core includes very simple data models which, abstractly, have immutable data and metadata, have methods to interrogate or transform the data in some fashion, and finally can read and write itself on disk without loss of precision. The TFBeam is an example of such an object and is the Python analog to the MATLAB “struct” containing a time-frequency reconstruction (NUTMEGs *beam* structure).

The toolbox side of NUTMEG-Py currently includes a nonparametric statistical testing package, based on Nichols and Holmes [55], including cluster level analysis from Hayasaka and Nichols [56]. Both approaches have been adapted to the five-dimensional space of time-frequency MEG imaging. The results are encapsulated in an object oriented manner, as the TimeFreqSnPMResults, which stores the generated null distributions, and has methods available for creating thresholds and maps based on levels of significance.

### 8.2. Visualization

**Figure 15:**
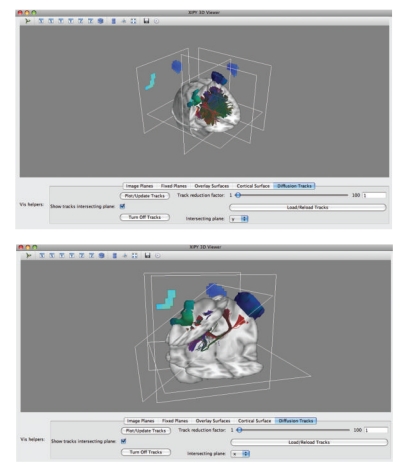
NUTMEG and DTI results in Xipy viewer.

While the MATLAB/SPM based visualization of results in NUTMEG allows for easy navigation across space, time, and frequency, the interactive viewing is limited to the orthogonal slice projection, which can make wide-spread global brain activations difficult to visualize. The project to transition NUTMEG into a Python-based toolkit has also spawned a small but powerful visualization effort named Xipy (cross-modality imaging in Python), which lies under the umbrella of the seminal NiPy (Neuroimaging in Python) project (http://nipy.sourceforge.net/). The main ambition of Xipy is to provide a flexible and extensible system for displaying and navigating brain imagery from various data sources (e.g., anatomical MRIs, functional maps, and diffusion tracks) in the same 3D scene (see Figure 15). Xipy is designed to be independent from NUTMEG-Py, and visualization of results from NUTMEG and NUTMEG-Py within Xipy is enabled by a richly featured plugin contained in the NUTMEG-Py package.

## 9. NUTMEG's Future Directions

The future of NUTMEG is influenced by both the research priorities of the developers as well as requests from users.

At present, we intend to create more formal links with SPM8, FieldTrip, and Brainstorm. Specifically, as methods developers, we would like to import, view, and directly compare the Multiple Sparse Priors [57] from SPM8 with other source estimation methods included in NUTMEG; further, we would like to enable direct comparison within NUTMEG of Dynamic Causal Modelling (DCM) for M/EEG [58] with other metrics for functional connectivity. The advanced time-frequency analysis and viewing tools for sensor level data within FieldTrip can be useful to NUTMEG users for planning of further analysis in source space. NUTMEG should be able to display source level results computed in FieldTrip. The cluster-based and permutation test statistics for sensor and source space results implemented in FieldTrip would also be of benefit to be more formally linked to the NUTMEG format. Sensor selection via visual inspection is a highly developed tool within Brainstorm, the output of which could be imported to NUTMEG. Brainstorm also contains useful GUIs for dataset, trial-condition selection, and batch processing setup, which could be linked to NUTMEG via a conversion of MATLAB data structures.

As several methods for connectivity analysis have recently become available within NUTMEG and additional methods are planned for inclusion, a means to visually browse the results is needed beyond a simple extension of the current source-space viewer. The eConnectome package (http://econnectome.umn.edu/) already implements the computation and elaborate visualization of connectivity, to which we may link.

The fusion of multiple sensor types (MEG magnetometers and planar gradiometers, scalp EEG, and intracranial EEG) simultaneously recorded for source reconstruction is a compelling need, but is not yet considered directly straightforward or well established; NUTMEG and other open-source software packages would benefit greatly from further developments on this topic.

## 10. Conclusion

NUTMEG provides a full set of MATLAB-based open-source functions with which to compute neural source estimates and additional manipulations thereof, as well as a graphical interface to process and view results. It is linked (to varying degrees) to other open-source packages for processing steps which are better performed by those toolboxes. NUTMEG is flexible to inclusion of new methods at any stage and welcomes new users and developers.

##  Authors' Contribution

Sarang S. Dalal and Johanna M. Zumer contributed equally to the manuscript.

## Figures and Tables

**Figure 8:**
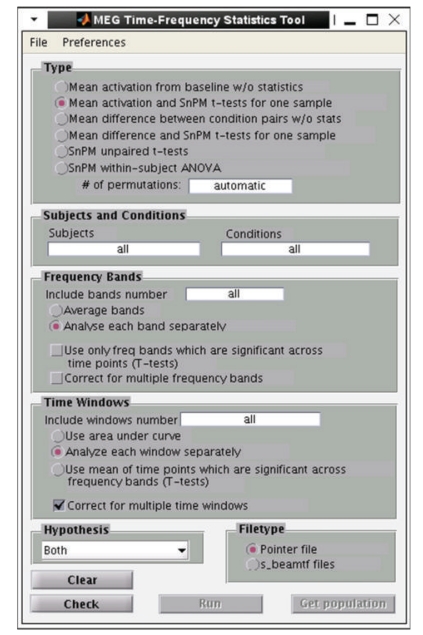
NUTMEGs time-frequency statistics tool, showing various options for calculating statistical significance across subjects for source time-frequency maps.

## References

[1] Dalal SS, Zumer JM, Agrawal V, Hild KE, Sekihara K, Nagarajan SS (2004). NUTMEG: a neuromagnetic source reconstruction toolbox. *Neurology & Clinical Neurophysiology*.

[2] Evans AC, Collins DL, Mills SR, Brown ED, Kelly RL, Peters TM 3D statistical neuroanatomical models from 305 MRI volumes.

[3] Mazziotta J, Toga A, Evans A (2001). A probabilistic atlas and reference system for the human brain: International Consortium for Brain Mapping (ICBM). *Philosophical Transactions of the Royal Society B*.

[4] Aguera P-E, Jerbi K, Caclin A, Bertrand O ELAN: A software package for analysis and visualization of MEG, EEG, and LFP signals.

[5] Dalal SS, Sekihara K, Nagarajan SS (2006). Modified beamformers for coherent source region suppression. *IEEE Transactions on Biomedical Engineering*.

[6] Dale CL, Findlay AM, Adcock RA (2010). Timing is everything: neural response dynamics during syllable processing and its relation to higher-order cognition in schizophrenia and healthy comparison subjects. *International Journal of Psychophysiology*.

[7] Heinks-Maldonado TH, Mathalon DH, Houde JF, Gray M, Faustman WO, Ford JM (2007). Relationship of imprecise corollary discharge in schizophrenia to auditory hallucinations. *Archives of General Psychiatry*.

[8] Zumer JM, Nagarajan SS, Krubitzer LA, Zhu Z, Turner RS, Disbrow EA (2010). MEG in the macaque monkey and human: distinguishing cortical fields in space and time. *Brain Research*.

[9] Dalal SS, Guggisberg AG, Edwards E (2008). Five-dimensional neuroimaging: localization of the time-frequency dynamics of cortical activity. *NeuroImage*.

[10] Zumer JM, Brookes MJ, Stevenson CM, Francis ST, Morris PG (2010). Relating BOLD fMRI and neural oscillations through convolution and optimal linear weighting. *NeuroImage*.

[11] Guggisberg AG, Dalal SS, Findlay AM, Nagarajan SS (2008). High-frequency oscillations in distributed neural networks reveal the dynamics of human decision making. *Frontiers in Human Neuroscience*.

[12] Van Wassenhove V, Nagarajan SS (2007). Auditory cortical plasticity in learning to discriminate modulation rate. *Journal of Neuroscience*.

[13] Hinkley LBN, Nagarajan SS, Dalal SS, Guggisberg AG, Disbrow EA (2011). Cortical temporal dynamics of visually guided behavior. *Cerebral Cortex*.

[14] Guggisberg AG, Honma SM, Findlay AM (2008). Mapping functional connectivity in patients with brain lesions. *Annals of Neurology*.

[15] Zumer JM, Brookes MJ, Stevenson CS, Morris PG, Shinkareva SV Oscillatory power and connectivity changes in a word decision task measured with MEG.

[16] Wong DDE, Gordon KA (2009). Beamformer suppression of cochlear implant artifacts in an electroencephalography dataset. *IEEE Transactions on Biomedical Engineering*.

[17] Dalal SS, Baillet S, Adam C (2009). Simultaneous MEG and intracranial EEG recordings during attentive reading. *NeuroImage*.

[18] Sarvas J (1987). Basic mathematical and electromagnetic concepts of the biomagnetic inverse problem. *Physics in Medicine and Biology*.

[19] Huang MX, Mosher JC, Leahy RM (1999). A sensor-weighted overlapping-sphere head model and exhaustive head model comparison for MEG. *Physics in Medicine and Biology*.

[20] Spinelli L, Andino SG, Lantz G, Seeck M, Michel CM (2000). Electromagnetic inverse solutions in anatomically constrained spherical head models. *Brain Topography*.

[21] Zhu Z, Disbrow EA, Zumer JM, McGonigle DJ, Nagarajan SS (2007). Spatiotemporal integration of tactile information in human somatosensory cortex. *BMC Neuroscience*.

[22] Van Veen BD, Van Drongelen W, Yuchtman M, Suzuki A (1997). Localization of brain electrical activity via linearly constrained minimum variance spatial filtering. *IEEE Transactions on Biomedical Engineering*.

[23] Sekihara K, Nagarajan SS, Poeppel D, Marantz A, Miyashita Y (2001). Reconstructing spatio-temporal activities of neural sources using an MEG vector beamformer technique. *IEEE Transactions on Biomedical Engineering*.

[24] Cheyne D, Bakhtazad L, Gaetz W (2006). Spatiotemporal mapping of cortical activity accompanying voluntary movements using an event-related beamforming approach. *Human Brain Mapping*.

[25] Brookes MJ, Zumer JM, Stevenson CM (2010). Investigating spatial specificity and data averaging in MEG. *NeuroImage*.

[26] Popescu M, Popescu EA, Chan T, Blunt SD, Lewine JD (2008). Spatio-temporal reconstruction of bilateral auditory steady-state responses using MEG beamformers. *IEEE Transactions on Biomedical Engineering*.

[27] Quraan MA, Cheyne D (2010). Reconstruction of correlated brain activity with adaptive spatial filters in MEG. *NeuroImage*.

[28] Prendergast G, Johnson SR, Hymers M, Woods W, Green GGR (2011). Non-parametric statistical thresholding of baseline free MEG beamformer images. *NeuroImage*.

[29] Serada M, Zumer JM, Brookes MJ, Adjamian P (2010). Modulation of AEF amplitude with frequency of tone stimulation.

[30] Nagarajan SS, Attias HT, Hild KE, Sekihara K (2007). A probabilistic algorithm for robust interference suppression in bioelectromagnetic sensor data. *Statistics in Medicine*.

[31] Zumer JM, Attias HT, Sekihara K, Nagarajan SS (2007). A probabilistic algorithm integrating source localization and noise suppression for MEG and EEG data. *NeuroImage*.

[32] Zumer JM, Attias HT, Sekihara K, Nagarajan SS (2008). Probabilistic algorithms for MEG/EEG source reconstruction using temporal basis functions learned from data. *NeuroImage*.

[33] Wipf DP, Owen JP, Attias HT, Sekihara K, Nagarajan SS (2010). Robust Bayesian estimation of the location, orientation, and time course of multiple correlated neural sources using MEG. *NeuroImage*.

[34] Pascual-Marqui RD (2002). Standardized low-resolution brain electromagnetic tomography (sLORETA): technical details. *Methods and Findings in Experimental and Clinical Pharmacology*.

[35] Dale AM, Liu AK, Fischl BR (2000). Dynamic statistical parametric mapping: combining fMRI and MEG for high-resolution imaging of cortical activity. *Neuron*.

[36] Kumihashi I, Sekihara K (2010). Array-gain constraint minimum-norm spatial filter with recursively updated gram matrix for biomagnetic source imaging. *IEEE Transactions on Biomedical Engineering*.

[37] Sekihara K, Sahani M, Nagarajan SS (2005). A simple nonparametric statistical thresholding for MEG spatial-filter source reconstruction images. *NeuroImage*.

[38] Singh KD, Barnes GR, Hillebrand A (2003). Group imaging of task-related changes in cortical synchronisation using nonparametric permutation testing. *NeuroImage*.

[39] Varela F, Lachaux JP, Rodriguez E, Martinerie J (2001). The brainweb: phase synchronization and large-scale integration. *Nature Reviews Neuroscience*.

[40] Friston KJ (2001). Brain function, nonlinear coupling, and neuronal transients. *Neuroscientist*.

[41] Nunez PL, Srinivasan R, Westdorp AF (1997). EEG coherency I: statistics, reference electrode, volume conduction, Laplacians, cortical imaging, and interpretation at multiple scales. *Electroencephalography and Clinical Neurophysiology*.

[42] Gross J, Kujala J, Hämäläinen M, Timmermann L, Schnitzler A, Salmelin R (2001). Dynamic imaging of coherent sources: studying neural interactions in the human brain. *Proceedings of the National Academy of Sciences of the United States of America*.

[43] De Pasquale F, Della Penna S, Snyder AZ (2010). Temporal dynamics of spontaneous MEG activity in brain networks. *Proceedings of the National Academy of Sciences of the United States of America*.

[44] Martino J, Honma SM, Findlay AM Resting functional connectivity in patients with brain tumors in eloquent areas.

[45] Nolte G, Bai OU, Wheaton L, Mari Z, Vorbach S, Hallett M (2004). Identifying true brain interaction from EEG data using the imaginary part of coherency. *Clinical Neurophysiology*.

[46] Brookes MJ, Hale J, Zumer JM Measuring functional connectivity in default mode network using hilbert envelope correlation.

[47] Darvas F, Ermer JJ, Mosher JC, Leahy RM (2006). Generic head models for atlas-based EEG source analysis. *Human Brain Mapping*.

[48] Jerbi K, Ossandón T, Hamamé CM (2009). Task-related gamma-band dynamics from an intracerebral perspective: review and implications for surface EEG and MEG. *Human Brain Mapping*.

[49] Ball T, Kern M, Mutschler I, Aertsen AD, Schulze-Bonhage A (2009). Signal quality of simultaneously recorded invasive and non-invasive EEG. *NeuroImage*.

[50] Chang N, Gulrajani R, Gotman J (2005). Dipole localization using simulated intracerebral EEG. *Clinical Neurophysiology*.

[51] Yvert B, Fischer C, Bertrand O, Pernier J (2005). Localization of human supratemporal auditory areas from intracerebral auditory evoked potentials using distributed source models. *NeuroImage*.

[52] Fuchs M, Wagner M, Kastner J (2007). Development of volume conductor and source models to localize epileptic foci. *Journal of Clinical Neurophysiology*.

[53] Korzyukov O, Pflieger ME, Wagner M (2007). Generators of the intracranial P50 response in auditory sensory gating. *NeuroImage*.

[54] Zhang Y, van Drongelen W, Kohrman M, He B (2008). Three-dimensional brain current source reconstruction from intra-cranial ECoG recordings. *NeuroImage*.

[55] Nichols TE, Holmes AP (2002). Nonparametric permutation tests for functional neuroimaging: a primer with examples. *Human Brain Mapping*.

[56] Hayasaka S, Nichols TE (2004). Combining voxel intensity and cluster extent with permutation test framework. *NeuroImage*.

[57] Friston K, Harrison L, Daunizeau J (2008). Multiple sparse priors for the M/EEG inverse problem. *NeuroImage*.

[58] Daunizeau J, Kiebel SJ, Friston KJ (2009). Dynamic causal modelling of distributed electromagnetic responses. *NeuroImage*.

